# A Scoping Review of School-Based Nutrition Education Interventions in the Islamic Republic of Iran

**DOI:** 10.1177/10598405251345078

**Published:** 2025-05-22

**Authors:** Basil H. Aboul-Enein, Stephen Gambescia, Teresa Keller, Nada Benajiba, Patricia J. Kelly

**Affiliations:** 1 College of Arts & Sciences, Health & Society Program, 14709 University of Massachusetts Dartmouth, North Dartmouth, MA, USA; 2 Faculty of Public Health and Policy, London School of Hygiene & Tropical Medicine, London, UK; 3 College of Nursing and Health Professions, 6527 Drexel University, Philadelphia, PA, USA; 4 School of Nursing, 4423 New Mexico State University, Las Cruces, USA; 5 Joint Research Unit in Nutrition and Food, RDC-Nutrition AFRA/IAEA, 108308 Ibn Tofail University-CNESTEN, Rabat, Kenitra, Morocco; 6 College of Nursing, Thomas Jefferson University, Philadelphia, PA, USA

**Keywords:** school, nutrition, Iran, child health

## Abstract

This scoping review aims to identify evidence-based school nutrition interventions for implementation in Iran's dynamic economic, cultural, and socio-political environment. A review of published studies (2004-August 2024) using the PRISMA-ScR guidelines across 14 databases was conducted. The efficacy of various interventions reported significant positive changes at multiple levels: anthropometric, biological, and knowledge, attitudes, and behaviors related to healthy eating and lifestyle. Investigators carrying out research on “what works” in school-based nutrition education and general healthy lifestyle programs and services in the Islamic Republic of Iran have a host of challenges; however, much more research needs to be done. While there are few studies in this area of inquiry, outcomes on the nutrition interventions to date are impressive. The major challenges in improving school-based nutrition programs include the education of young women, establishing national standards for school food programs and education, and strategic advocacy by stakeholders to improve nutrition education in schools in Iran.

## Introduction

The World Health Organization (WHO) has identified childhood overweight and obesity as an ongoing concern in most developing countries (WHO, 2024). School nutrition is a pressing issue that influences the health and well-being of students and shapes their future into adulthood. Children with healthy dietary choices and behaviors are less likely to suffer from obesity, undernutrition, and physical developmental issues (Omidvar et al., 2021; UNICEF., 2019). The Middle East, including Iran, is grappling with a double burden of both undernutrition (stunting and wasting) and overnutrition (obesity/overweight) among school-age populations (Minaie et al., 2019; Mohseni et al., 2022; Motlagh et al., 2011; Sanjari et al., 2023; Sotoudeh et al., 2021; UNICEF., 2019; WHO Regional Office for the Eastern, 2022; World Bank., 2022; Zavoshy et al., 2012). This complex problem has become a critical public health issue for Iran as its economy grows and living standards improve.

The World Bank classified Iran as a developing upper-middle-income country with natural gas, petroleum, and agricultural resources that support economic growth (World Bank., 2022, 2024). One consequence of this economic growth are dietary changes present across the country, with individuals and families moving from traditional eating patterns to Westernized diets that feature energy-dense and processed foods (Hojhabrimanesh et al., 2017; Mirmiran et al., 2016), This Western diet, characterized by high consumption of fats, sugars, and processed ingredients, has been a major driver of childhood obesity and overweight, as well as an important contributor to an increase in non-communicable diseases, such as heart disease and diabetes (Armoon & Karimy, 2019; Floor, 2021; Hojhabrimanesh et al., 2017; Mirmiran et al., 2016; Ronto et al., 2018; Sanjari et al., 2023).

Improving school nutrition is an important strategy for instilling healthy eating habits in students (Bevans et al., 2011; Saavedra & Prentice, 2023; WHO, 2021b). The US Centers for Disease Control (CDC) suggests that evidence-based strategies optimizing school nutrition programs include establishing nutritional standards; educating students, teachers, staff, families, and communities about healthy nutrition; and limiting access to non-nutritional foods in the school environment (CDC, 2024). WHO recommendations are consistent with CDC recommendations, and include the need to subsidize healthy foods for schoolchildren in regions where prices for these foods might be out of reach for school districts (WHO, 2021a).

In Iran, the central government has taken steps to improve school nutrition by developing the Healthy School Canteen Program (HSC) targeting school canteens as centers of nutrition service upgrades (Babashahi et al., 2021). However, since the implementation of this program in 2011, evaluation studies have found serious implementation problems (Babashahi et al., 2021; Omidvar et al., 2021; Yazdi-Feyzabadi et al., 2018), including a lack of clear nutritional standards, economic issues resulting from high food prices, poor nutritional literacy among students and school staff, limited availability of professional support from qualified nutritionists, and poorly prepared canteen personnel (Ahmadi & Karamitanha, 2023; Amerzadeh et al., 2023; Doustmohammadian et al., 2019; Minaie et al., 2019; Yazdi-Feyzabadi et al., 2018). In some cases, conflicts of interest among participating stakeholders impede the efficient distribution of food and effective operation of school canteens (Babashahi et al., 2021; Omidvar et al., 2022).

The socio-cultural values of Iranian families and communities further complicate the implementation of national school nutrition programs (Razi & Nasiri, 2022). Studies with families of schoolchildren found issues of parental conflict and indecisiveness, and children's stubbornness and secretiveness to be perceived as influences on children's food intake. Socially, the entire family and even neighbors have input into the parenting of children and their nutritional input, a reflection of the traditional cohesive view of families as the center of decision-making (Razi & Nasiri, 2022). Family beliefs about health and body image can also hinder the effectiveness of school nutrition programs, with many elderly Iranians believing that obesity is a sign of good health (Razi & Nasiri, 2022). At the community level, a distinct lack of support for childhood obesity programs has been identified (Niknam et al., 2021; Niknam et al., 2023). Other parents identified the influence of a political and sociocultural context that did not support girls having an active lifestyle, an important contributor to obesity (Mohammadpour-Ahranjani et al., 2014).

School nutrition is also greatly affected by large national and international geopolitical forces. Economic sanctions imposed by the US and its allies in response to the Iranian nuclear program have aggravated both food price inflation and food insecurity for Iranian households (Hejazi & Emamgholipour, 2022). Coupled with a drop in the value of Iranian currency, these political and economic forces have resulted in an increase in food-vulnerable Iranian children (Mohammadi-Nasrabadi et al., 2023; RezaeeDaryakenari et al., 2024; UNICEF., 2024; Zamanialaei et al., 2023).

Given these challenges, the need to find practical, culturally congruent school nutrition programs to assist Iranian schoolchildren and families to avoid malnutrition is critical. This scoping review aimed to identify feasible, evidence-based school nutrition interventions for implementation in Iran's dynamic economic, cultural, and socio-political environment.

## Methods

### Selection Criteria

The Population, Intervention, Comparison, Outcomes, and Study (PICOS) design guidelines (Higgins et al., 2019) were incorporated to develop the research question: “*Do* *school-age students* *in Iran (P) that are offered school-based nutrition interventions (I) have improved health and wellness parameters (O) compared with those that do not participate in school-based nutrition interventions(C)?”* and the subsequent inclusion and exclusion criteria (see Table 1). Peer-reviewed articles published in English or Farsi were included. Interventions reported outside of traditional peer-reviewed articles were excluded from this review. The search was conducted in the summer of 2024, and the results communicate the literature published between 2004 and August 2024.

**Table 1.** PICOS Criteria for Inclusion and Exclusion of Studies.

Parameter	Inclusion Criteria	Exclusion Criteria
Population	Kindergarten through Grade 12 School-aged students (i.e., around 6 years old and above), who were examined in Iran within the school setting	Students who are not of school-age. Students who are not studying in Iran Students undergoing medical nutrition therapy-based diets Preschool children
Intervention type	Any kind of school-based intervention that addresses nutrition-related aspects, including Educational interventions Environmental Interventions Multi-componential Interventions	Interventions that are not based on school facilities Interventions that do not address nutrition-related outcomes
Comparators	Pre-intervention, baseline nutrition-related variables (i.e., anthropometric measures, biochemical parameters, nutrition-related knowledge, dietary habits, perceived hunger) of student groups who were: Control: received no intervention. Received partial intervention e.g., educational intervention only vs. multicomponential intervention	N/A
Outcomes of Interest	Changes in anthropometric outcomes, e.g.,: BMI for age, height for age changes in biochemical outcomes changes in nutrition-related knowledge changes in meeting the dietary macronutrient and/or micronutrient recommendations changes in adherence to healthy dietary habits and avoidance of unhealthy ones changes in risks of nutrition-related diseases e.g.,: obesity or iron-deficiency anemia changes in short-term hunger	Non-nutrition related outcomes
Language	English or Farsi	All other languages
Study type	Peer-reviewed original research articles Original research conference publications Intervention-based studies	Non peer-reviewed articles Commentaires Narratives Protocols Communications Nonintervention-based studies White papers Similar article types Gray literature Theses/dissertations

BMI = body mass index; N/A = not applicable.

### Search Procedures

A scoping review of the literature was conducted using Arksey and O’Malley's methodological framework (Arksey & O'Malley, 2005) and PRISMA Extension for Scoping Reviews (Tricco et al., 2018). We began a comprehensive search within biomedical databases using a combination strategy of medical subject heading keywords, terms, phrases, and Boolean operators (see the Supplemental Material). The following 14 databases were searched: EBSCOHost, BIOSIS, CINAHL, ScienceDirect, ArticleFirst, Biomed Central, BioOne, ProQuest, SAGE Reference Online, Scopus, SpringerLink, PubMed, Taylor and Francis, and Wiley Online. The search strategies were adapted according to the indexing systems of each database (see the Supplemental Material).

### Study Selection and Data Extraction

**Figure 1.**
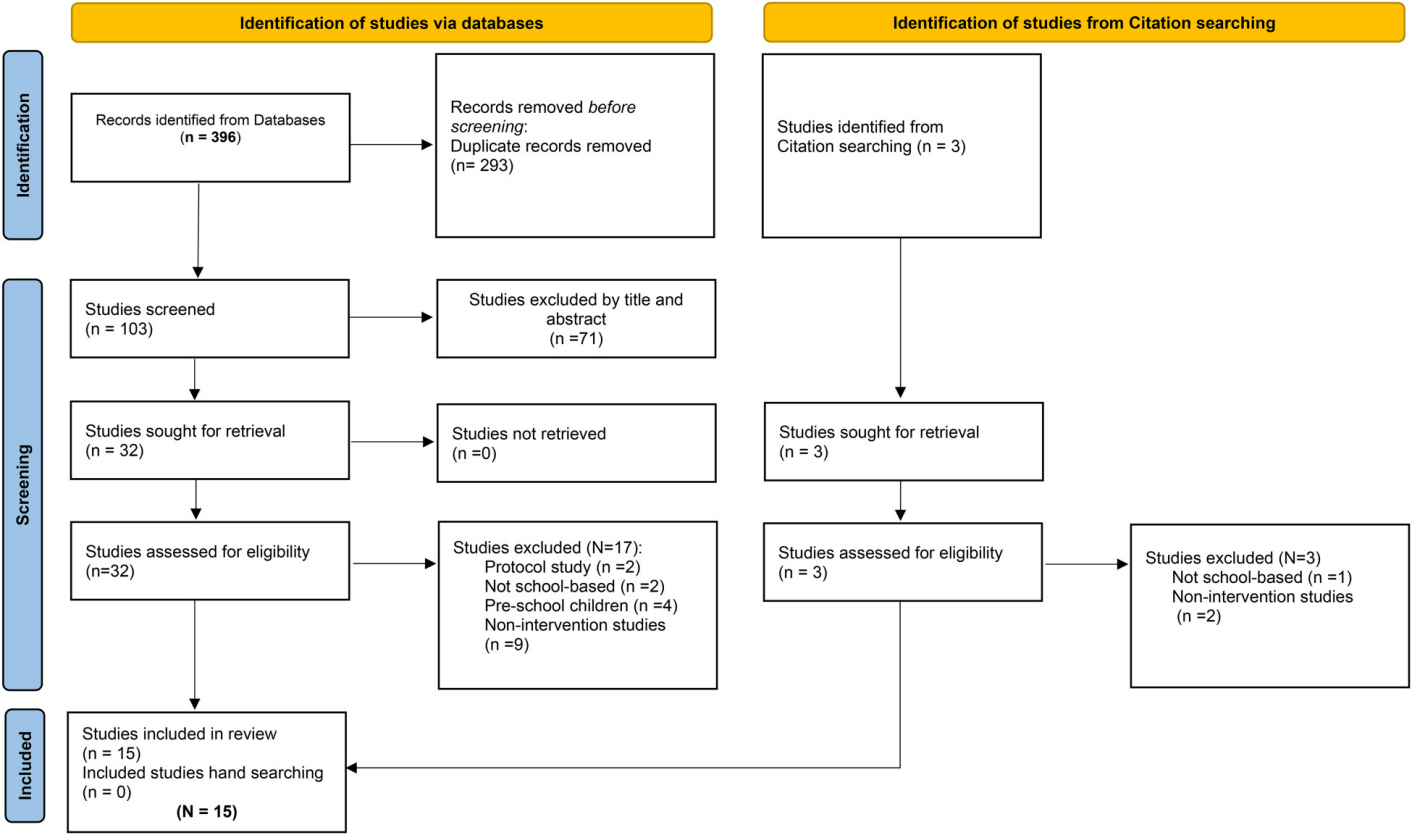
Search flow diagram following PRISMA 2020 guidelines.

Two of the authors searched for relevant articles, and one author used Rayyan QCRI software (Ouzzani et al., 2016) to assist in the screening process. All retrieved articles were screened for relevance to the topic (Figure 1). In addition, reference lists from the retrieved articles were manually reviewed to identify any additional relevant publications. Titles and abstracts were screened for relevance, and potentially relevant journal abstracts were reviewed by four authors. Potential articles included in this review were evaluated for relevance, merit, and inclusion/exclusion criteria (see Table 1). The articles accepted for inclusion were reviewed individually by each author. In addition, the reference list of each included article was screened for potentially eligible articles. Once the list of selected studies was finalized, two authors extracted and cross-checked each study. One author updated the search, reviewed the articles, and wrote the first draft of the results and discussion section of the review. Differences in opinions in the extracted data were discussed to reach a consensus and tabulated (see Table 2). As methodological quality assessment is not a prerequisite for scoping reviews, we did not appraise the included studies (Peters et al., 2020).

**Table 2.** Intervention Characteristics of Included Studies (N = 15).

**Author, Year**	**Goal**	**Design**	**Participants**	**Intervention Content**	**Theoretical Framework**	**Results**	**Discussion Points**
**Ahmadpour et al., 2023**	Design, implement, and evaluate a FNLIT food and nutrition literacy promotion intervention	RCT 3 phase study, intervention development, implementation x 6 months, evaluation	300 (IG: 150/ CG:150) 10–12-year-old students;	Nutrition education to parents/school staff, students; environmental interventions to create a healthy food environment at home and school	Intervention mapping	Significant difference between the sig & CG scores in all subscales of FNLIT (*p* < .001).	Intervention provides the basis for future programs targeting the improvement of FNLIT in children, especially in poor and deprived areas
**Amini et al., 2016**	Evaluate the effect of intervention to reduce excess weight gain in primary school-age children	Cluster RCT over 18 weeks	334 (IG: 167/ CG:167) overweight or obese 4^th^-6^th^ grade students	Nutrition education, increased physical activity (PA) for pupils, lifestyle modification for parents, and changing food items sold at the school canteen	N/R	Significantly reduced BMI-Z-score (*p* = .003), hip circumference (*p* < .001); IG vigorous PA, energy intake increased (*p* < .001); triceps skinfold thickness, computer time not changed in IG, increased in CG (*p* < .001; *p* = .004)	Study provides a model for implementation of similar interventions
**Amini et al., 2023**	Assess the long-term effect of school-based intervention for weight reduction	13-month f/up of cluster RCT	59% (205) of students completed f/up			BMI-Z-score decreased in both IG and CG, but the change was more significant in. IG (*p* = .003); a more significant effect on girls’ scores than boys (*p* < .001)	Intervention is an effective way to lose weight; the effect lasted for a relatively long time
**Fathi et al., 2017**	Determine the effect of nutrition education on reducing consumption of unhealthy snacks	2-group pre-post intervention assessment	88 6^th^ grade girls (IG: 44; CG: 44)	45-min education sessions, which included mothers and teachers	Health Belief Model	Scores of all variables and behavior of unhealthy snacks consumption significantly increased in IG (*p* < .05).	HBM nutrition education program effective in reducing consumption of unhealthy snacks, increased scores of HBM constructs, decreased scores of perceived barriers
**Haghani et al., 2017**	Determine the effects of tailored education on lifestyle modification in elementary school students	Quasi-experimental RCT followed at 1- and 4-month postintervention	64 students from 2 schools (IG:32; CG: 32),	4 60-min weekly education sessions provided by a health education specialist	N/R	Average nutrition and physical activity scores had significant differences between groups (*p* < .001).	Providing overweight students with education improves their general lifestyle, reduces weight; recommend application with overweight children
**Hosseini et al., 2015**	Increase breakfast consumption	Cluster RCT, 2 months post intervention assessment	88 children from 4 schools	5 sessions of collaborative learning	Theory of Reasoned Action	IG breakfast scores significantly increased *p* < .01); increase in all nutritional intakes except fat & sugar (*p* < .01)	Recommend applying theory to increase breakfast eating
**Jadgal et al., 2020**	Examine the effects of peer education on behavioral nutrition	Quasi-experimental design	160 female 4^th^ grade female students	Peer education program	Theory of Planned Behavior	Knowledge& behavior change in IG greater than CG (*p* < .001; *p* < .001); significant change in IG perceived behavioral control (*p* < .001)	Education based on theory & training of peers effective in positively effecting behavior
**Jeihooni et al., 2021**	Evaluate the effectiveness of PRECEDE model of nutrition education on iron deficiency anemia	Quasi-experimental design with 4 month f/up	160 f7th-8^th^ grade female students	six sessions based PRECEDE model for 45 or 50 min	PRECEDE Model	IG had a greater increase in theory variables, knowledge, & ferritin levels	PRECEDE model intervention improved iron deficiency anemia preventive behaviors
**Joulaei et al., 2013**	Assess the effects of a community-nutrition intervention	Cluster RCT x 2 years	2897 7–13-year-old students from 3 school	Expand the National Free Food Program to low-income areas; substitute nutritious snacks in public schools; advocacy actions	Public health advocacy	Post-intervention, IG girls had 5% lower BMI (*p* = .02); no significant changes among boys; IG & CG knowledge increased *p* < .0001).	Demonstrates scalability of school feeding programs in Iran; nutrition reduced the prevalence of underweight
**Nabipour et al., 2004**	Assess if a 1-year classroom-based education can change knowledge scores about a healthy heart	RCT with 14 schools x 8 weeks	1128 3^rd^ & 4^th^ grade students	2 h/week sessions on heart function, nutrition, exercise for a healthy heart, no tobacco	N/R	Posttest, IG knowledge than CG (*p* < .001)	Schools provide excellent setting for healthy heart education
**Rouhani-Tonekaboni et al., 2023**	Change dairy consumption of female students based on TTM	Pre-post quasi-experimental study	159 female students (IG: 56; CG:103) in 10-11th grades	IG of groups of 8-9 students received 4 45-min sessions, content on student stages of change in dairy consumption	TTM	Mean scores of behavioral change processes, cognitive change processes, decisional balance, self-efficacy improved in IG (*p* < .05 for all)	Study showed that intervention based on the TTM can positively affect dairy consumption behaviors
**Shahraki-Sanavi et al., 2018**	Investigate the effect of school-based interventions on health-risk behaviors among adolescents	Quasi-experimental intervention x 9 months	420 adolescent females	Multidimensional interventions focused on nutrition, physical activity, substance use, sex behaviors (individual & group education, parent education; counseling	N/R	Post-intervention, three simultaneous behaviors decreased among IG by 8.4% compared to 1.6% in CG	School-based interventions can improve health behaviors if target environmental/ behavioral dimensions
**Shirazi et al., 2019**	Evaluate intervention on dietary behaviors & behavioral determinants	RCT	230 13–15-year-old girls (IG: 4115; CG: 4115)	Multicomponent intervention packages for adolescents, parents, teachers.	SCT	Changes in most behavioral determinants are significantly associated with dietary behavior changes	Intervention based on SCT effective in improving dietary behaviors, but behaviors not at ideal status
**Soheila et al., 2016**	Assess the effect of nutrition education on students’ knowledge, attitude, & performance	2 group pre-post quasi experimental design	180 primary school students (IG: 90; CG: 90)	Intervention was 5 45-min classes with principles of nutrition, food pyramid, nutrition during school, snacks, common nutrition problems	N/R	Significant difference in pre-post knowledge, attitude, performance (*p* < .05)	Nutritional education may improve knowledge, attitude and performance of primary school students
**Toulabi et al., 2012**	Determine the influence of “behavior modification” program on BMI in obese public high school students	RCT	152 adolescents	Intervention was education, modifying dietary habits, exercise program 3 days/week, teaching parents nutrition,	Behavior Modification	Adolescent's mean weight, BMI, waist/ hip circumferences in IG decreased after program (*p* < .001).	Program effective in reducing BMI in obese students; suggest principals consider implementing program

BMI = body mass index; BMI-Z = body mass index Z-score; CG = control group; FNLIT = food and nutrition literacy; N/R = not reported; IG = intervention group; SCT = social cognitive theory; TTM = transtheoretical model.

## Results

### Characteristics of the Studies

The initial search resulted in N = 396 articles. After eliminating duplicates and screening titles and abstracts for eligibility, we identified 32 full-text papers, of which 15 satisfied the inclusion criteria. The publications ranged from 2004 to 2023, peaking in 2023 with four publications. This systematic review included studies examining students from 4th to 11th-12th grades, thereby encompassing both children and adolescents. Notably, one study focused on overweight and obese students (Amini et al., 2016). Among the 15 studies, 9 included participants of both sexes, while 6 specifically targeted female participants (Fathi et al., 2017; Jadgal et al., 2020; Jeihooni et al., 2021; Rouhani-Tonekaboni et al., 2023; Shahraki-Sanavi et al., 2018; Shirazi et al., 2019). The sample sizes varied significantly, ranging from 64 (Haghani et al., 2017) to 2897 (Joulaei et al., 2013). Seven studies employed a design that divided participants into control and intervention groups (Ahmadpour et al., 2023; Amini et al., 2016; Fathi et al., 2017; Haghani et al., 2017; Rouhani-Tonekaboni et al., 2023; Shirazi et al., 2019; Soheila et al., 2016). The duration of the interventions varied considerably, from 2 months (Hosseini et al., 2015; Nabipour et al., 2004) to 2 years (Joulaei et al., 2013), with the majority of interventions being less than one year in length. In six studies, the duration of the intervention was not indicated (Fathi et al., 2017; Jadgal et al., 2020; Rouhani-Tonekaboni et al., 2023; Shirazi et al., 2019; Soheila et al., 2016; Toulabi et al., 2012).

### Study Design

The majority of the studies included in this review (N = 9) utilized randomized controlled trials (RCTs), with four of these being cluster RCTs. Five studies employed a quasi-experimental design: Jadgal et al. (2020); Jeihooni et al. (2021); Rouhani-Tonekaboni et al. (2023); Shahraki-Sanavi et al. (2018); Soheila et al. (2016). Additionally, four articles used a pre- and post-intervention design (Fathi et al., 2017; Haghani et al., 2017; Rouhani-Tonekaboni et al., 2023; Soheila et al., 2016). Notably, three studies incorporated follow-up assessments into their methodologies (Amini et al., 2023; Jeihooni et al., 2021).

### Intervention Characteristics (Content and Framework)

More than half of the interventions included in this review (N = 8) were based on theoretical frameworks or health-promotion models. Each study employed a specific model, which is as follows: intervention mapping (Ahmadpour et al., 2023), health belief model (Fathi et al., 2017), theory of reasoned action (Hosseini et al., 2015), theory of planned behavior (Jadgal et al., 2020), PRECEDE model (Jeihooni et al., 2021), public health advocacy (Joulaei et al., 2013), transtheoretical model (Rouhani-Tonekaboni et al., 2023), and social cognitive theory (Shirazi et al., 2019). Additionally, one intervention focused specifically on behavioral modification.

The interventions employed in this study were designed to enhance health outcomes through a multifaceted approach incorporating educational sessions, collaborative learning, and peer education programs. Most interventions are multicomponent, integrating nutrition with other lifestyle factors, including physical activity (Amini et al., 2016), substance use, and sexual behaviors (Haghani et al., 2017), as well as cardiovascular health and tobacco cessation (Nabipour et al., 2004). Several studies have focused on interventions involving staff and parents, highlighting the importance of a comprehensive approach (Ahmadpour et al., 2023; Amini et al., 2016; Amini et al., 2023; Fathi et al., 2017; Shahraki-Sanavi et al., 2018; Shirazi et al., 2019). Among the reviewed literature, four studies reported that the duration of educational sessions was consistently 45 min (Fathi et al., 2017; Rouhani-Tonekaboni et al., 2023; Soheila et al., 2016). This structured timeframe may enhance the engagement and retention of information, contributing to the overall effectiveness of interventions.

### Outcomes

The analysis of the efficacy of various interventions reported in the 15 studies included in this literature review showed significant positive changes at multiple levels: anthropometric, biological, knowledge, attitudes, and behaviors related to healthy eating and lifestyle.

**Biological outcomes:** One study exclusively assessed the impact of the intervention on the biological outcomes (Joulaei et al., 2013). The authors found that the intervention group experienced a significantly greater increase in ferritin levels than the control group, highlighting the potential effectiveness of the intervention in improving iron status.

**Anthropometric outcomes:** Several studies reported a significant reduction in the BMI Z-score (Amini et al., 2023; Jeihooni et al., 2021; Toulabi et al., 2012), as well as a decrease in hip circumference (*p* < 0.01) (Amini et al., 2023) and waist-to-hip ratio (Toulabi et al., 2012) in the intervention group compared to the control group. These findings were also highlighted by Amini et al. (2023).

**Knowledge and behavioral changes:** Several studies have demonstrated significant improvements in dietary behavior as a result of this intervention. Notably, increased breakfast scores were reported by Hosseini et al. (2015) along with enhanced nutritional intake observed in multiple studies (Haghani et al., 2017; Hosseini et al., 2015; Shirazi et al., 2019). Additionally, Fathi et al. (2017) noted a reduction in unhealthy snacking behaviors. Furthermore, Fathi et al. (2017) documented improvements in general behavioral outcomes (Rouhani-Tonekaboni et al., 2023; Shahraki-Sanavi et al., 2018; Shirazi et al., 2019; Haghani et al., 2017). These findings underscore the intervention's positive impact on various aspects of dietary habits and overall behavior.

With regard to nutrition knowledge, Jadgal et al. (2020) demonstrated a significant increase in this parameter following intervention. Similar findings have been reported by Jeihooni et al. (2021), Joulaei et al. (2013), Nabipour et al. (2004), and Soheila et al. (2016). These studies collectively highlight the effectiveness of the intervention in enhancing the participants’ understanding of nutrition. Additionally, improvements were observed in perceived behavioral control following the intervention (Jadgal et al., 2020). Jeihooni et al. (2021), whereas Rouhani-Tonekaboni et al. (2023) noted increases in self-efficacy. Soheila et al. (2016) documented positive changes in attitudes and performance. Taken together, these findings suggest the efficacy of the intervention in fostering multiple aspects of behavioral change.

## Discussion

Investigators carrying out research on “what works” in school-based nutrition education and general healthy lifestyle programs and services in the Islamic Republic of Iran have a host of challenges. Such challenges range from the bi-modal nature of the health status among school-age children from unhealthy eating by mimicking the diets of developing countries and the consequent risk of being overweight and obese, to undernourishment of young people in low-income families. While children have a culture of support in their upbringing, extended family members with a hand in rearing and neighbors, and the larger community involved, such broad influence creates tension, conflicts in nutrition messaging, and inconsistency in developing healthy food routines, and presumably healthy behaviors. Also influencing obesity outside of family and school nutrition programs, are environmental barriers limiting physical activity in Iranian children (Kelishadi et al., 2010). Ideally, family and school influences on both nutrition and physical activity should be in sync to prevent obesity among youth.

At the macro level, the steady geopolitical life in Iran results in instability in commitment from government officials to school health programs and support, economic challenges, family and school funding, public health officials’ ability to implement programming and services with high fidelity, and to diffuse any best practices to schools across the country. However, despite the changing economic, cultural, and socio-political environment in Iran, there are impressive indicators that research efforts in this area are on the right track. For example, the diversity of theories used in these 15 school-based nutrition intervention studies during this almost 20-year period of review bodes well that in time signature and branded nutrition education programs that are evidence-based are likely to emerge. Investigators are aware of and use in good form a line of classic health behavior theories (Nutbeam et al., 2022), such as the health belief model, theory of reasoned action, theory of planned behavior, transtheoretical model, and social cognitive theory. Another positive finding from this review is that broader and progressive models of school nutrition education are evident in the use of the PRECEDE model of health promotion and health advocacy (Carlisle, 2000; Green et al., 2022). Given the concerns regarding multiple stakeholders and the lack of school food program standards to improve youth nutrition, Mohammadpour-Ahranjani et al. (2014) stated that a prominent emerging theme was the need for state-level intervention and support for a healthy environment. Any local initiatives in Iran are unlikely to be successful without such support (Mohammadpour-Ahranjani et al., 2014, p. 83).

Investigators are using acceptable research designs, such as high-standard randomized controlled trials (9 of 15) and control groups, even when using convenience sampling. Pre- and post-test designs are common in identifying useful outcomes in school health education programs; however, a longer duration of both students’ exposure to lessons and follow-up assessments should be an objective for future research. Investigators have used a range of types of instructors delivering lessons or programs to improve youth's nutrition knowledge and behavior, from nutritionists to health educators, to a promising peer education program, as seen in the study by Jadgal et al. (2020). Program development should favor tools that can be used by on-site teachers to maximize the reach of students efficiently. Therefore, training programs for schoolteachers at all levels should be prioritized in future evaluations.

Other findings in this scoping review show that, while the results of only 15 studies were published in this almost 20-year period, there is diversity in the specific aims, research design, age/grades of student participants (K-12), content, and use of recognized theories. Hopefully, more work will be done in this area of inquiry, and investigators will receive support from stakeholders. An impressive finding from this review is the positive reports of the important outcomes of the specific aims of the projects. The descriptors “significant” and “greater” are used to describe efforts to improve knowledge acquisition and retention of good nutrition, and changes in behaviors that resulted in positive changes in biomarkers and anthropometric markers (BMI-Z-Scores, decrease in hip circumference, and waist-to-hip ratio). Although not always measured by participants in a study, the interventions focus on the importance of maintaining an ideal weight. Investigators in Iran during this period have examined the main areas of improving nutrition among school students: knowledge, attitudes, and behavioral practices, and each study has encouraged major change to gain state support for healthy nutrition policies.

## Implications for School Nurses, Health Policy, Practice, and Equity

− More research is needed in this important area to improve the lives of school-age children and adolescents in Iran, given that only 15 studies have been published in peer-reviewed journals during this almost 20-year period.
− The national government should consider school nutrition as a priority to counter the bimodal challenge of both under- and overnutrition.
− Educating Iranian mothers and women of childbearing age is one of the most significant variables in healthy children.
− Calls were made by investigators to develop a national school nutrition program supported by all stakeholders, including standards for what needs to be taught, by whom, and attention to setting the scope and sequence of content. This is important given the people's changes in food consumption habits as the nation is developing and thus getting the youth “on the right track” in healthy eating and regular physical activity.
− Work done to date in this area has impressive behavior change outcomes, with appropriate inputs of theory-driven projects with appropriate research designs to examine the range of student age/grade levels. More can be done to learn about or report on instructors delivering nutrition education and summative evaluations of program delivery.
− Efforts can be made to develop evidence-based “branded” nutrition programs that can be diffused throughout the schools.
− Strategic advocacy by school administrators, teachers, school nurses, health educators, nutritionists, health professionals, and parents is crucial for improving school nutrition education programs in Iran.
− Lastly, the applicability of school nutrition policies may be extended beyond Iran to aid Islamic schools in other Muslim-predominant countries and communities by encouraging Islamic schools to implement culturally and religiously congruent healthful dietary guidelines.

## Conclusion

The use of diverse health behavior theories and good forms in a range of research designs across a sampling of ages/grades of Iranian school students bodes well that, in time, signature and branded nutrition education programs that are evidence-based are likely to emerge. The efficacy of various interventions showed significant positive changes at multiple levels: anthropometric, biological, and knowledge, attitudes, and behaviors related to healthy eating and lifestyle. Investigators carrying out research on “what works” in school-based nutrition education and general healthy lifestyle programs and services in the Islamic Republic of Iran have a host of challenges, such as high inflation, bimodal youth health status of overweight and obesity with significant youth undernourished, dynamic socio-cultural and political changes, status of women affected by socio-cultural factors/barriers who serve as children's first health educators, lack of government standards for food programs sponsored by school, and what needs to be taught in schools. Strategic advocacy and culturally congruent approaches by all stakeholders are crucial for improving school nutrition education programs in Iran.

## Supplemental Material

sj-docx-1-jsn-10.1177_10598405251345078 - Supplemental material for A Scoping Review of School-Based Nutrition Education Interventions in the Islamic Republic of Iran Supplemental material, sj-docx-1-jsn-10.1177_10598405251345078 for A Scoping Review of School-Based Nutrition Education Interventions in the Islamic Republic of Iran by Basil H. Aboul-Enein, Stephen Gambescia, Teresa Keller, Nada Benajiba and Patricia J. Kelly in The Journal of School Nursing
