# Supplementary material for: A Scoping Review of School-Based Nutrition Education Interventions in the Islamic Republic of Iran
Source: J Sch Nurs. 2025 May 22;42(1):19–31. doi: 10.1177/10598405251345078 (PMC12819892; doi:10.1177/10598405251345078)
Supplement: sj-docx-1-jsn-10.1177_10598405251345078 - Supplemental material for A Scoping Review of School-Based Nutrition Education Interventions in the Islamic Republic of Iran [file sj-docx-1-jsn-10.1177_10598405251345078.docx]

**Supplementary material**

“School” [All Fields]; “school-based” [All Fields]; “elementary” [All Fields]; Primary” [All Fields]; “Secondary” [All Fields]; “nutrition” [All Fields]

AND

“Intervention” [All Fields]; “Program” [All Fields]; “Education” [All Fields]

AND

“Iran” [All Fields]
